# Immunogenic relationship mapping supports a minimal-set trivalent vaccine strategy for broad sarbecovirus protection

**DOI:** 10.1038/s41392-025-02565-5

**Published:** 2026-02-09

**Authors:** Yeqing Sun, Ziqi Cheng, Xi Wu, Yunbo Bai, Lina Zhao, Hongyu Xiang, Weijin Huang, Jianhui Nie

**Affiliations:** 1 School of Life Sciences, Jilin University, Changchun, China; 2 Division of HIV/AIDS and Sex-transmitted Virus Vaccines, National Institutes for Food and Drug Control, State Key Laboratory of Drug Regulatory Science, NHC Key Laboratory of Research on Quality and Standardization of Biotech Products, NMPA Key Laboratory for Quality Research and Evaluation of Biological Products, Beijing, China

**Keywords:** Vaccines, Vaccines

## Abstract

Major outbreaks of severe acute respiratory syndrome (SARS) and coronavirus disease 2019 (COVID-19), together with the continuous risk of zoonotic spillover of animal sarbecoviruses, underscore the urgent need for vaccines that confer broad protection across the sarbecovirus subgenus. Current immunogen selection strategies for pansarbecovirus vaccine development predominantly rely on phylogenetic or spike sequence conservation analyses, which often fail to accurately predict the breadth of cross-neutralization. To overcome this limitation, we systematically evaluated cross-neutralization profiles among 25 representative sarbecoviruses from clades 1 and 3 via guinea pig antisera individually raised against full-length spike proteins in pseudovirus neutralization assays while excluding clade 2 viruses lacking known receptor usage. Neutralization profiling revealed four distinct immunogenic clusters that diverged from traditional phylogenetic relationships. Antisera induced by the palm civet–derived SARS-CoV-1 strain SZ1 broadly neutralized all clade 1a viruses, whereas full coverage of clade 1b viruses required at least two distinct immunogens. Remarkably, sera elicited by multiple clade 1 immunogens also neutralized clade 3 viruses despite no prior exposure to clade 3 antigens. Guided by these findings, we proposed a minimal trivalent immunogen combination—SZ1, SARS-CoV-2, and PCoV-GX—that elicited broad neutralization against both clade 1 and clade 3. This rational approach eliminates the need for additional clade 3–specific antigens and provides a preclinical framework for developing next-generation pansarbecovirus vaccines.

## Introduction

Over the past two decades, the sarbecovirus subgenus within the β-coronavirus family has caused two major outbreaks of zoonotic infections in humans: the severe acute respiratory syndrome coronavirus (SARS-CoV) outbreak in 2002–2003 and the coronavirus disease 2019 pandemic caused by severe acute respiratory syndrome coronavirus 2 (SARS-CoV-2) from 2019–2023.^1–3^ Additionally, some animal-origin coronaviruses, such as the Wuhan Institute of Virology 1 (WIV1), *Rhinolophus sinicus* SHC014 (RsSHC014), *Rhinolophus affinis* TG13 (RaTG13), Pangolin coronavirus Guangdong (PCoV-GD), and Pangolin coronavirus Guangxi (PCoV-GX), can infect cells via the use of human angiotensin-converting enzyme 2 (ACE2) as a receptor.^4–9^ These animal-origin coronaviruses have the potential to cross species barriers and cause disease in humans, thereby increasing the likelihood of other large-scale coronavirus outbreaks in the future. Thus, there is an urgent need to develop pansarbecovirus vaccines that can prevent zoonotic sarbecovirus infections.

A series of broad-spectrum anti-sarbecovirus or even pancoronavirus vaccines have been developed to combat existing virus strains. For example, mosaic (mRBD-NP) and cocktail (cRBD-NP) nanoparticle vaccines were created by combining equimolar amounts of the receptor-binding domains (RBDs) of SARS-CoV-2, SARS-CoV-1, WIV1, and RaTG13 either within nanoparticles or as separate components.^10^ Another example is the pansarbecovirus vaccine Mosaic-8b, which was designed with nanoparticles that present the beta RBD of SARS-CoV-2 along with the RBDs of seven other sarbecoviruses (RaTG13, RsSHC014, *Rhinolophus sinicus* 4081 (Rs4081), Pangolin coronavirus strain 17 (Pang17), *Rhinolophus malayanus* YN02 (RmYN02), *Rhinolophus ferrumequinum* 1 (Rf1), and WIV1)).^11^ Regardless of vaccine design, the initial selection and design of antigens play critical roles. Currently, antigen selection for pansarbecovirus vaccines often relies on the phylogenetic distances of spike proteins or RBDs, the conservation of neutralizing antibody epitopes, and structural features.^11,12^ Whereas some evidence suggests that phylogenetic distance is correlated with antigenic similarity, genetic and immunological relationships between coronaviruses have been noted,^13–15^ highlighting the need for experimental validation of cross-neutralization patterns. Currently, no systematic cross-neutralization studies have compared immunogenic relationships across all major sarbecovirus clades to guide rational vaccine design. This knowledge gap limits our ability to select minimal immunogen sets for maximum protective breadth. Therefore, the immunogenicity and antigenic relationships among sarbecovirus strains must be elucidated to guide the design and development of pansarbecovirus vaccines.

Therefore, this study aimed to (1) systematically delineate cross-neutralization profiles across 25 representative sarbecoviruses; (2) uncover immunogenic clustering relationships that deviate from conventional phylogenetic classifications; and (3) define a minimal, yet broadly protective, immunogen set capable of eliciting cross-sarbecovirus immunity. Collectively, these efforts seek to inform rational antigen design and accelerate the development of next-generation pansarbecovirus vaccines.

## Results

### Selection of sarbecovirus strains for immunization

**Fig. 1:**
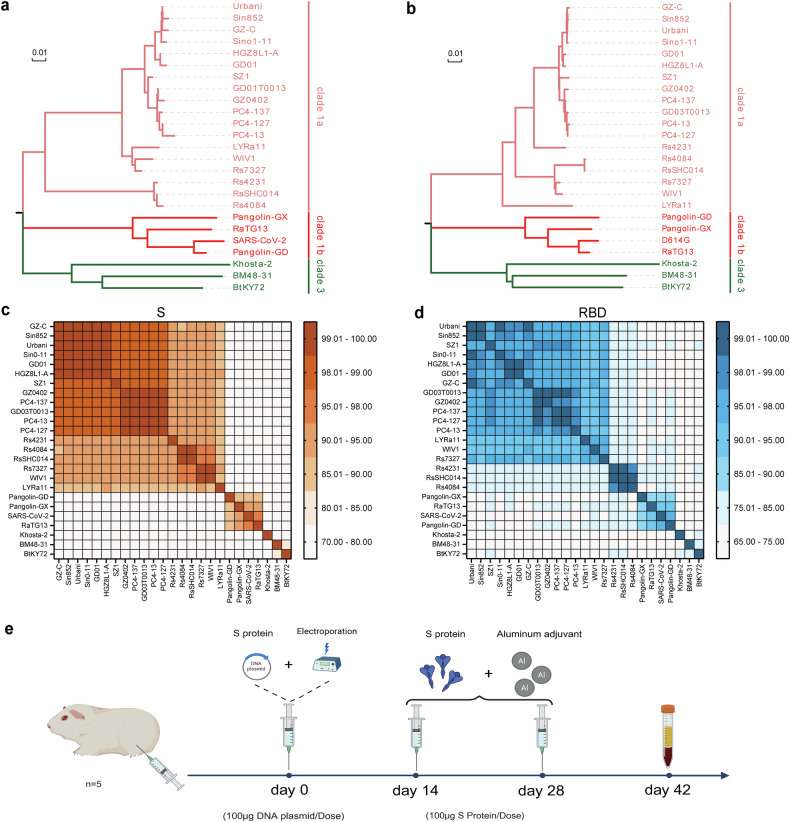
Selection and analysis of 25 sarbecovirus immunogens. **a**, **b** Phylogenetic trees of sarbecovirus S proteins and RBD regions constructed from amino acid sequences. **c**, **d** Heatmaps showing the amino acid sequence identities of the Sarbecovirus S proteins and RBD regions. **e** Immunization protocol diagram. Guinea pigs were immunized following a DNA prime–protein boost regimen. The initial DNA immunization (100 μg of spike-encoding plasmid DNA) was delivered via intramuscular injection followed by electroporation. Two booster doses containing 100 μg of the corresponding purified S protein formulated with an equal volume of aluminum adjuvant were administered at 14-day intervals after priming. Blood samples were collected 14 days after the third immunization for serum preparation

This study assessed the cross-neutralization activity of sarbecoviruses via a pseudovirus neutralization assay. The initial strain selection was guided by the phylogenetic framework established by Starr et al.^16^, which comprehensively delineated the known ACE2-utilizing sarbecoviruses (Supplementary Fig. 1). Because the cellular receptor for clade 2 viruses remains unidentified and no suitable titration cell lines are available, clade 2 members were excluded from this analysis. To ensure broad phylogenetic and host diversity while maintaining experimental feasibility, we selected 25 representative sarbecoviruses from clades 1a, 1b, and 3. The selection encompassed strains that cover distinct phylogenetic sublineages within each ACE2-using clade and differ in host species (bats, civets, pangolins, and humans). Phylogenetic trees were subsequently constructed on the basis of the amino acid sequences of the full-length S protein and its RBD for these selected viruses (Fig. 1a and b). The topologies inferred from the S- and RBD-based trees were largely concordant, although subtle differences were observed within clade 1a and, more prominently, among clade 1b viruses. In particular, several clade 1b strains (e.g., SARS-CoV-2, RaTG13, and PCoV-GD) presented relatively shifted positions between the two trees, suggesting that distinct evolutionary pressures act on the RBD region. The amino acid identities of the S proteins between SARS-CoV and SARS-like coronaviruses in clade 1a exceeded 87.4% (Fig. 1c), whereas the RBD identity was greater than 80.7% (Fig. 1d). The S protein sequence identities between clade 1a and clades 1b and 3 ranged from 75.7 to 78.0% and from 72.6 to 76.0%, respectively (Fig. 1c). The corresponding RBD identities were 72.6–77.6% and 69.0–75.3%, respectively (Fig. 1d). In clades 1b and 3, the S protein sequence identities ranged from 71.4 to 72.3%, and the RBD identities ranged from 66.4 to 73.5%. Notably, S protein sequence identities were consistently greater than those of the RBD. Among clade 1b strains, the full-length amino acid sequence identities of S proteins between SARS-CoV-2 and RaTG13 and between SARS-CoV-2 and PCoV-GD were 97.3% and 89.9%, respectively; the RBD amino acid sequence identities were 90.1% and 96.9%, respectively (Fig. 1c and d). These results indicate that SARS-CoV-2 shares greater overall S protein similarity with RaTG13 than with PCoV-GD does, although its RBD is more similar to that of PCoV-GD than to that of RaTG13.

To investigate the antigenic clustering of immune responses among various sarbecovirus lineages, we immunized guinea pigs with the spike (S) proteins of 25 representative sarbecovirus strains. A DNA prime–protein boost regimen was used, as this heterologous immunization strategy has been shown to enhance both germinal center formation and affinity maturation, resulting in stronger and broader neutralizing antibody responses than homologous immunization schemes.^17–22^ The initial immunization consisted of plasmids encoding the full-length S protein DNA, which was administered intramuscularly followed by electroporation to increase DNA uptake, followed by two booster immunizations with the corresponding purified S proteins formulated with an aluminum adjuvant on days 14 and 28 after priming. Serum samples were collected two weeks after the third immunization for cross-neutralization analysis (Fig. 1e). To ensure comparability among immunogens, all 25 Sarbecovirus S DNA plasmids were confirmed to exhibit comparable in vitro expression levels (Supplementary Fig. 2). The corresponding purified S proteins used for booster immunizations exhibited greater than 95% purity (Supplementary Fig. 3).

### Comparison of the cross-neutralization reactivity of guinea pig sera monovalently immunized with pseudoviruses from 25 sarbecovirus strains

We evaluated the cross-neutralizing activity of guinea pig sera against 24 sarbecovirus pseudoviruses (excluding BM48-31, which lacks receptor usage). Because sarbecoviruses differ substantially in their utilization of TMPRSS2 and other host proteases for cell entry,^23,24^ pseudovirus neutralization assays were performed using 293T-hACE2 cells to maintain assay consistency and ensure cross-lineage comparability.

**Fig. 2:**
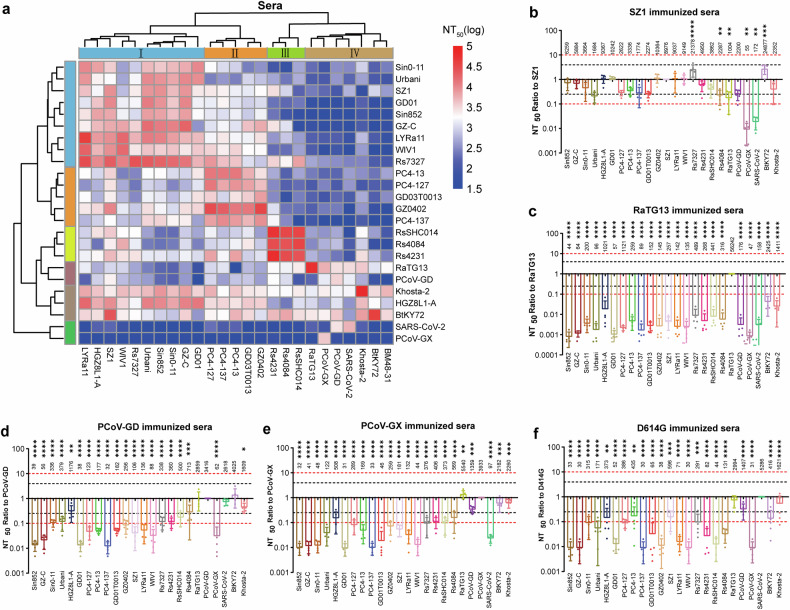
Cross-neutralization analysis of sera from guinea pigs immunized with a monovalent DNA prime and two doses of spike protein corresponding to 25 different sarbecoviruses, tested against pseudoviruses from 24 sarbecovirus strains. **a** Clustered heatmap of log-transformed NT50 values from cross-neutralization assays of guinea pig sera against 24 sarbecovirus pseudoviruses. The values represent the mean titers from five animals per immunogen group; both axes are hierarchically clustered to depict immunogenic and antigenic relationships. **b**–**f** The x-axis shows the sarbecovirus pseudovirus strains, and the y-axis shows the NT50 values relative to those of the immunogen-matched strains. The black and red dashed lines denote 4-fold and 10-fold differences, respectively. Each dot represents one guinea pig (the mean of three technical replicates; *n* = 5 per group). The results are presented as the means ± standard deviations (SDs). Two-way ANOVA and Dunnett’s multiple comparisons test were used for statistical analysis. Significant differences compared with the homologous immunization group are indicated by asterisks

We analyzed the immunogenic relationships among sarbecoviruses via an antigenic cartography-based approach, in which immunogenic relationships were defined as cross-neutralization patterns among immune sera and antigenic relationships were represented as quantitative distances derived from pseudovirus neutralization assays. By comparing logarithmic NT50 values, we determined the antigenic distances between serum–virus pairs. To objectively define group boundaries, Ward’s hierarchical clustering was applied to the antigenic distance matrix, revealing four major clusters (Fig. 2a).

Cluster I includes SARS-CoV-1 strains (Sin852, GZ-C, Sino1-11, Urbani, HGZ8L1-A, and GD01) along with three bat coronaviruses (LYRa11, WIV1, and Rs7327). Cluster II consists of PC4-127, PC4-13, PC4-137, GD03T0013, and GZ0402. Cluster III includes Rs4231, RsSHC014, and Rs4084. Notably, evolutionary clades 1b (SARS-CoV-2, PCoV-GD, PCoV-GX, and RaTG13) and 3 (BM48-31, BtKY72, and Khosta-2) exhibited similar antigenic and immunogenic profiles and were thus grouped into a single cluster (Cluster IV).

In clade 1a, SZ1 stood out as the broadest immunogen, effectively neutralizing all clade 1a pseudoviruses with only modest reductions in titer (Fig. 2b). Other SARS-CoV-1 strains and related bat or civet strains segregated into two additional groups, which is consistent with distinct antigenic relationships despite close sequence similarity (Supplementary Fig. 4a–q).

Within clade 1b, RaTG13 induced potent autologous responses but showed little cross-neutralization, whereas sera from SARS-CoV-2 D614G, PCoV-GD, and PCoV-GX demonstrated broader activity across clade 1b, although with moderate reductions in titer (Fig. 2c–f). To further dissect homologous and cross-neutralization patterns within clade 1b, we compared reciprocal pseudovirus neutralization among RaTG13, PCoV-GD, PCoV-GX, and SARS-CoV-2 (Supplementary Fig. 5a–d). RaTG13 sera showed strong homologous neutralization (NT₅₀ ≈ 5.6 × 10⁴) but weak or undetectable activity against SARS-CoV-2 and PCoV strains, with >40-fold reductions. Conversely, sera raised against SARS-CoV-2 or PCoV strains partially neutralized RaTG13 (NT₅₀ ≈ 3 × 10³–5 × 10³), indicating asymmetric cross-reactivity within clade 1b. These findings support the distinct antigenic position of RaTG13 and highlight the directional nature of antibody cross-recognition among sarbecoviruses.

Two clade 3 sarbecoviruses, BtKY72 and Khosta-2, presented extremely low or undetectable entry efficiency in human ACE2-expressing cells (Supplementary Fig. 6a). To obtain measurable and biologically interpretable neutralization readouts, pseudovirus assays for these two strains were performed using the ACE2 orthologs that each virus can efficiently utilize (Rhinolophus affinis ACE2 for BtKY72 and rabbit ACE2 for Khosta-2).

Importantly, this choice was made solely for methodological consistency rather than to imply natural host receptor usage. Previous studies have shown that the ACE2 compatibility of sarbecoviruses often does not correlate with the host species from which the viruses were isolated and that cross-species receptor usage is highly heterogeneous across subgenera.^16,25,26^ Using permissive ACE2 orthologs ensures accurate NT₅₀ determination for strains with extremely poor entry through human ACE2, enabling a valid comparison across the full pseudovirus panel. The immune sera from BtKY72, Khosta-2, and other strains strongly cross-neutralized each other (NT_50_ ≥ 3000) (Supplementary Fig. 6b–d). Surprisingly, sera raised against many clade 1 immunogens also neutralized clade 3 pseudoviruses, revealing unanticipated cross-clade antigenic relationships.

Collectively, these findings highlight the antigenic heterogeneity within clades 1a and 1b, the broad neutralizing capacity of SZ1, and the unexpected cross-reactivity between clade 1 and clade 3 sarbecoviruses.

### Amino acid residues affecting sarbecovirus antigenicity

**Fig. 3:**
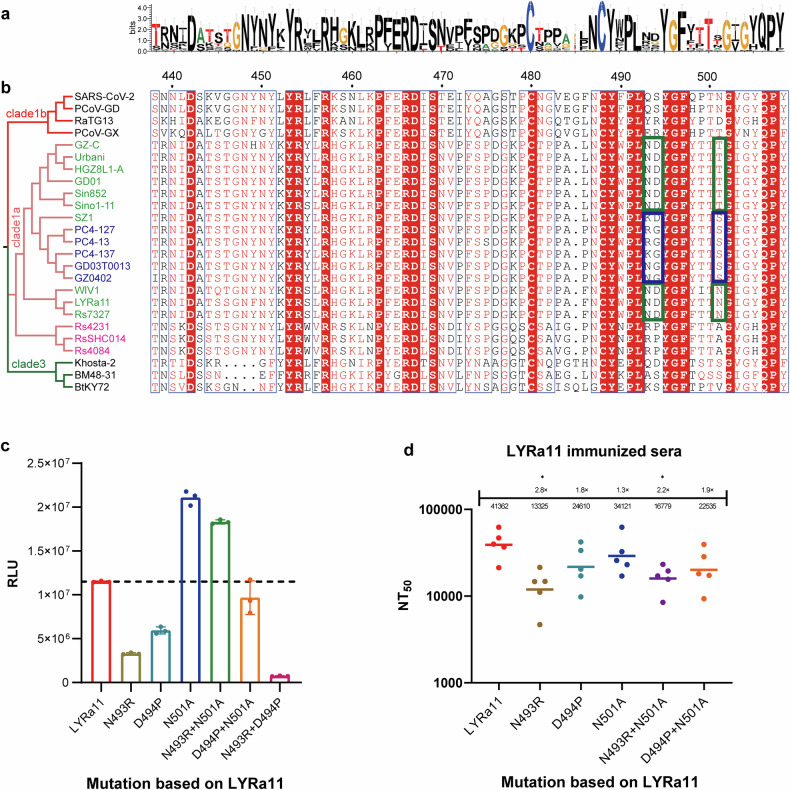
Analysis of amino acid residues affecting sarbecovirus antigenicity. **a** Sequence conservation of the RBM region among 25 sarbecoviruses. The number of SARS-CoV-2 residues is indicated. **b** Phylogenetic tree constructed from the RBD amino acid sequences of 25 sarbecoviruses, along with multiple sequence alignments of RBM regions. Different phylogenetic clades are highlighted in distinct colors. Strains with similar antigenic profiles are shown in the same color, and RBM sequence regions associated with antigenicity are boxed in corresponding colors. **c** LYRa11 was selected as a representative strain; its spike protein residues at positions 493, 494, and 501 were individually or doubly mutated to the corresponding amino acids in RsSHC014. Pseudovirus titration was performed using these LYRa11 mutants. **d** Pseudovirus neutralization assays using LYRa11 immune sera against wild-type LYRa11 and its mutants. Each dot represents the NT_50_ value of an individual guinea pig serum sample. The data are presented as the means of three independent experiments. The bars indicate the means ± standard deviations

The RBD is the primary target of sarbecovirus neutralization, with major sequence variations concentrated in the receptor-binding motif (RBM). To explore whether specific amino acid residues shape antigenic properties, we compared RBM sequences and antigenic profiles across clade 1a viruses (Fig. 3a and b). Rs4231, RsSHC014, and Rs4084 share only 52% RBM amino acid identity with other clade 1a viruses, corresponding to their distinct antigenic profiles. In contrast, although PC4-127, PC4-13, PC4-137, GD03T0013, and GZ0402 display high overall RBD identity (91–93%) with Sin852, GZ-C, Sino1-11, Urbani, HGZ8L1-A, and GD01, their RBM identity (91–93%) is slightly lower than that of LYRa11, WIV1, and Rs7327 (93–94%) (Figs. 1d, 3b). These comparisons indicate that positions 493, 494, and 501 represent major sites of divergence within the RBM.

We selected LYRa11 as the template for mutagenesis because it is closely related to WIV1 and Rs7327—sharing 93–94% RBM amino acid identity—but differs at key residues (493, 494, and 501), making it an ideal representative background for probing the functional effects of these substitutions. Single- and double-point mutations were introduced at the corresponding positions, replacing the LYRa11 residues with those present in RsSHC014, which exhibits distinct antigenicity. Mutant pseudoviruses were successfully constructed and titrated to equivalent concentrations for infectivity and neutralization assays. Infectivity testing in 293T-ACE2 cells revealed that most mutants presented reduced entry efficiency relative to that of wild-type LYRa11, with the N493R + D494P mutant completely losing infectivity—likely due to structural instability or impaired ACE2 binding (Fig. 3c). Neutralization assays further demonstrated that while most mutants retained pseudovirus neutralization titers similar to those of wild-type LYRa11, the N493R single mutant and N493R + N501A double mutant partially escaped neutralization (2–3-fold reduction), highlighting the role of these residues in modulating antigenicity (Fig. 3d).

### Immunogenic relationships among sarbecoviruses

**Fig. 4:**
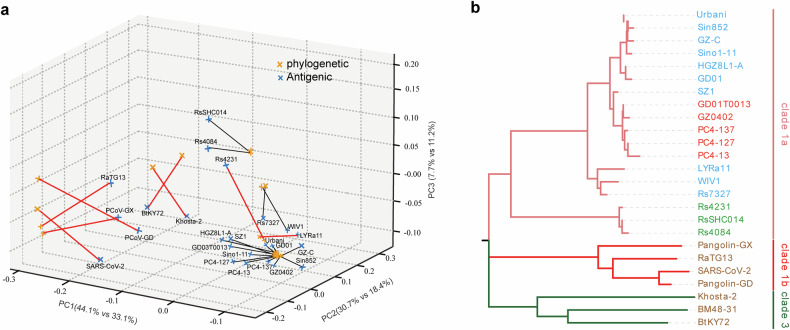
Immunogenic relationships among sarbecoviruses. **a** Phylogenetic and antigenic distance matrices were independently subjected to principal coordinate analysis (PCoA) and subsequently aligned via Procrustes transformation. The blue and red points denote phylogenetic and antigenic coordinates, respectively, with lines connecting paired positions of the same strain. The black lines indicate general concordance, whereas the eight strains with the largest displacements are highlighted with bold red lines and labels, representing viruses for which genetic relatedness and antigenic similarity diverge most strongly. **b** Comparison of RBD amino acid sequence phylogenetic relationships with immunogenic relationships. Strains with similar immunogenicity are shown in the same color

We then performed three-dimensional PCoA on both the antigenic and phylogenetic distance matrices (Supplementary Fig. 7). To directly compare sequence-based relatedness with empirically measured antigenic relationships, we aligned the 3D PCoA embeddings of the two matrices via Procrustes analysis (Fig. 4a). This analysis indicated moderate but significant global concordance (Mantel *r* = 0.685, *p* < 0.001; Procrustes *r* = 0.679) while also revealing a measurable mismatch between the two spaces (disparity = 0.539). Many clade 1a viruses formed similar neighborhoods in both maps, which is consistent with their broadly concordant cross-neutralization patterns. In contrast, eight strains—including RaTG13, BtKY72, Khosta-2, and Rs4231—showed particularly large displacements, highlighting cases where genetic relatedness fails to predict antigenic similarity (Fig. 4b).

Together, these analyses clarify that antigenic clustering broadly aligns with phylogenetic relationships but also reveals nonlinear divergences driven by functional variations within the RBD. A quantitative comparison between the two spaces further demonstrated that empirical antigenic mapping provides a more functionally relevant and experimentally validated framework for immunogen selection.

Whereas phylogenetic grouping would require at least four representative strains to achieve partial lineage coverage—with residual gaps within clade 1b—the antigenic clustering derived from cross-neutralization data indicates that a minimal trivalent combination (SARS-CoV-1 SZ1, PCoV-GD, and SARS-CoV-2) achieves complete cross-neutralization across all 25 tested sarbecoviruses.

This outcome highlights the practical value of antigenic cartography in guiding parsimonious yet broadly protective vaccine design and provides a data-driven rationale for selecting the trivalent immunogen combination evaluated in subsequent experiments.

### Cross-neutralization reactivity between trivalent immunized guinea pig sera and sarbecoviruses

**Fig. 5:**
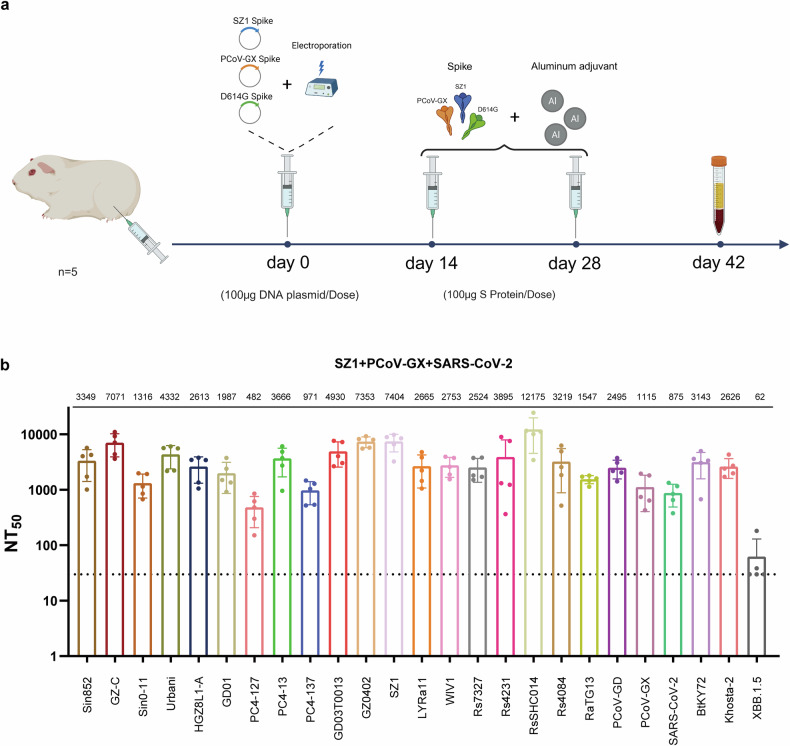
Cross-neutralization reactivity between trivalent immunized guinea pig sera and 25 sarbecovirus pseudoviruses. **a** Schematic of the trivalent immunization regimen and blood collection in guinea pigs. The regimen consisted of a priming dose with spike DNA, followed by two booster doses containing spike protein formulated with alum. Sera were collected 14 days after the final immunization. **b** Neutralization assays of trivalent immunized guinea pig sera against 25 sarbecovirus pseudoviruses. Immunogens are indicated in red. Each dot represents the NT_50_ value from an individual guinea pig serum sample. The data are presented as the means of three independent experiments. The bars indicate the means ± standard deviations

Because monovalent immunogens are unable to neutralize all 25 selected sarbecovirus strains and current pansarbecovirus vaccine development strategies rely primarily on multivalent formulations, we selected three immunogens—SARS-CoV-1 SZ1, SARS-CoV-2 D614G, and PCoV-GX—on the basis of the cross-neutralization results of monovalent immunization. Using a sequential immunization strategy that consisted of priming with spike protein DNA followed by protein boosters, guinea pigs received three doses; sera were collected 2 weeks after the final immunization (Fig. 5a). The collected sera were subjected to cross-neutralization assays against all 25 pseudovirus strains. The results demonstrated that sera from guinea pigs immunized with the trivalent vaccine exhibited broad-spectrum neutralizing activity (Fig. 5b). However, the trivalent immune sera did not neutralize the SARS-CoV-2 XBB.1.5 variant, which is consistent with expectations. This lack of neutralization is likely attributable to the substantial evolutionary distance between SARS-CoV-2 XBB variants and the D614G immunogen, as well as multiple mutations within the RBD that confer immune escape. These findings underscore the importance of carefully selecting vaccine immunogens to target SARS-CoV-2 variants, with particular attention given to the immunogenic relationships among variants. We also evaluated neutralizing antibody kinetics across the three-dose regimen. No measurable neutralizing activity was detected 14 days after DNA priming. Neutralization increased substantially after the first protein boost and reached the highest levels following the second boost, while maintaining a stable breadth profile (Supplementary Fig. 8). This study was conceived within the framework of pandemic preparedness rather than seasonal SARS-CoV-2 circulation. By mapping the antigenic relationships among diverse sarbecoviruses, we aimed to establish a broadly applicable reference for rational immunogen selection.

## Discussion

The emergence and global spread of SARS-CoV-1 and SARS-CoV-2 have drawn substantial attention to zoonotic sarbecoviruses and heightened concern regarding their potential to cause future outbreaks. These factors have accelerated efforts to develop broad-spectrum vaccines capable of countering SARS-CoV-1, SARS-CoV-2, and other potential zoonotic sarbecovirus threats.

Currently, most immunogen selection strategies for pansarbecovirus vaccine design rely on sequence conservation or phylogenetic analyses. However, sequence similarity does not necessarily correspond to the conservation of antigenic epitopes, and genetic distance does not always reflect antigenicity or immunogenicity. In this study, we immunized guinea pigs with monovalent vaccines via spike proteins from 25 representative sarbecoviruses, and then evaluated their serum neutralizing activity through vesicular stomatitis virus (VSV)-based pseudovirus neutralization assays. Except for BM48-31, for which no effective cell receptor has been identified, we successfully constructed 24 VSV-based sarbecovirus pseudoviruses.

Cross-neutralization assays revealed that the clustering relationships in sarbecovirus immunogenicity and antigenicity did not fully align with the ancestral phylogeny of the spike protein or RBD sequences (Fig. 2a). Within clade 1a, three immunogenic subclusters were identified:

Cluster I: Sin852, GZ-C, Sino1-11, Urbani, HGZ8L1-A, GD01, LYRa11, WIV1, and Rs7327;

Cluster II: PC4-127, PC4-13, PC4-137, GD03T0013, and GZ0402;

Cluster III: Rs4231, RsSHC014, and Rs4084.

These findings indicate that clade-based phylogenetic classification alone is insufficient for the precise design of immunogen combinations.

Notably, SZ1-induced immune sera in guinea pigs effectively cross-neutralized all clade 1a strains (Fig. 2b), highlighting its potential as a central immunogen. SZ1 is a SARS-CoV strain isolated from palm civets.^27^ Its spike protein and RBD share 98.4–98.8% and 97.3–98.6% sequence identity, respectively, with those of other SARS-CoV strains (Fig. 1c, d). Thus far, there have been no reports specifically characterizing the immunogenicity of SARS-CoV-1 SZ1.

Consistent with our previous findings, broad cross-neutralization was observed between SARS-CoV-2 and PCoV-GD but not between SARS-CoV-2 and PCoV-GX (Fig. 2c–f).^24^ Additionally, the immunogenicities of SARS-CoV-2 D614G, PCoV-GD, and PCoV-GX were significantly lower than those of SARS-CoV-1 and related clade 1a strains (Fig. 2a), likely owing to higher levels of spike protein glycosylation and a greater proportion of the closed RBD conformation.^28,29^ Our results also demonstrated that RaTG13 induced high-titer, strain-specific pseudovirus neutralization responses, but exhibited limited cross-neutralization against other clade 1 strains (Fig. 2c). In contrast, immune sera from other clade 1b strains effectively neutralized RaTG13 (Fig. 2a). These findings suggest that, when designing pansarbecovirus vaccines, at least two immunogens targeting clade 1b strains are necessary to achieve complete coverage of clade 1b viruses.

This study also revealed that immune sera induced by nearly all strains effectively neutralized pseudotyped Khosta-2 and BtKY72 (Fig. 2a). Both Khosta-2 and BtKY72 belong to clade 3; they exhibit similar antigenicity and immunogenicity. These results suggest that antibody responses showing strong pseudovirus neutralization activity against clade 1 viruses could also cross-neutralize clade 3 sarbecoviruses.

To investigate the relationship between RBM sequence variation and sarbecovirus antigenicity, we introduced single- and double-point mutations into LYRa11 and then evaluated their impact on antigenicity. The results showed that only the N493R mutation caused a two- to threefold reduction in neutralization, without complete antibody escape. This outcome is consistent with previous reports showing that broad pseudovirus neutralization activity is typically mediated by antibodies that target conformationally conserved multiresidue epitopes, particularly hydrophobic patches. Single- or even double-point mutations are often insufficient to disrupt antibody binding, whereas broader immune escape generally requires coordinated changes at multiple sites.^30–32^

Through cross-neutralization analysis of guinea pig sera immunized with a trivalent immunogen combination (SARS-CoV-1 SZ1, SARS-CoV-2 D614G, and PCoV-GX), we found that this immunogen set elicited potent and broad neutralizing activity against sarbecovirus strains, excluding SARS-CoV-2 variants.

In summary, by clustering sarbecoviruses on the basis of their antigenic and immunogenic profiles, we developed an antigenic cartography that can inform vaccine antigen selection, with the goal of identifying a minimal set of immunogens to achieve maximal protective breadth in future vaccine design. An important consideration in rational vaccine design is the balance between antigenic coverage and immune focus: excessively multivalent formulations may induce antibody competition or immunodominance biases, whereas minimal immunogen sets can maximize coverage while preserving response balance.

Our findings demonstrate that a trivalent combination (SARS-CoV-1 SZ1, PCoV-GD, and SARS-CoV-2) achieves broad cross-neutralization across major sarbecovirus lineages without unnecessary complexity, providing a preclinical framework for identifying minimal immunogen sets that balance breadth and focus.

While this study was based on pseudovirus assays in an animal model and certain SARS-CoV-2 variants and Clade 2 viruses were not evaluated, it establishes a foundational empirical map to guide subsequent in vivo validation of protection via live-virus challenge models. Future studies will extend this framework by incorporating variant-updated or mosaic immunogens and evaluating their protective efficacy against emerging SARS-CoV-2 variants and other sarbecoviruses in suitable animal systems. Together, our findings provide a preclinical foundation for guiding next-generation sarbecovirus vaccines that balance broad coverage with adaptability to viral evolution.

## Materials and methods

### Cells

293 T cells were purchased from the American Type Culture Collection (ATCC, Cat: CRL-3216). ACE2-expressing stable cell lines were established via lentiviral transduction and maintained in our laboratory.^33^ Briefly, cells derived from 293 T cells stably expressing human ACE2 were used in this study. The cells were cultured in Dulbecco’s modified Eagle’s medium (high glucose; HyClone) supplemented with 100 U/mL penicillin‒streptomycin (Gibco) and 10% fetal bovine serum (Pansera ES, PAN-Biotech) in a 5% CO_2_ atmosphere at 37 °C. The cells were passaged every 2–3 days via 0.25% trypsin-ethylenediaminetetraacetic acid (Gibco).

### Plasmid construction

Sequences encoding sarbecovirus spike proteins were codon optimized for human expression and cloned and inserted into the pcDNA3.1 vector. The sources and accession numbers of the 25 sarbecoviruses are summarized in Supplementary Table 1. In total, 12 mutant plasmids were generated on the basis of these constructs. Site-directed mutagenesis was performed via methods described in our previous studies.^34^ The specific mutation sites and corresponding primers (synthesized by China Biotechnology) are listed in Supplementary Table 2.

### Protein expression and purification

The proteins used in this study were provided by Acro Biosystems. The spike trimer, His-tagged and comprising the ectodomain of the sarbecovirus spike proteins, was expressed in human 293 cells (HEK293) with a T4 fibritin trimerization motif and a polyhistidine tag at the C-terminus. Proline and alanine substitutions were introduced to stabilize the trimeric prefusion state of the spike protein and abolish the furin cleavage site, respectively. Proteins were purified via a Ni affinity column and a Superdex 200 gel filtration column (GE Healthcare) and were quality checked via SDS–PAGE (>95%). The protein was delivered as a bulk protein in a 0.2 μm filtered solution of 0.1 M sodium citrate, pH 5.5, with 10% trehalose as a protectant and frozen at −80 °C until further use.

### Guinea Pig immunization and serum preparation

Female Hartley guinea pigs weighing 200–220 g (*n* = 5 per group) were immunized via three intramuscular injections into the thigh muscle, following a DNA prime–protein boost immunization strategy.^22,35^ The first dose consisted of 100 μg of spike plasmid DNA, which was administered via intramuscular injection immediately followed by electroporation (TERESA-A, TERESA) to increase in vivo DNA uptake and expression.^36^ The second and third doses, which were administered at 14-day intervals, contained 100 μg of the corresponding purified S protein mixed with an equal volume of aluminum adjuvant. The pcDNA3.1-derived expression vectors were subjected to both forward and reverse sequencing via the T7 promoter and bGH sequences present on the vector to ensure the fidelity of the inserted fragment. Fourteen days after the final immunization, blood samples were collected to extract the antisera. The animal study protocol was approved by the Animal Welfare and Ethics Review Committee of the National Institute for Food and Drug Control (Approval No. NIFDC number 2024(B)021).

### Production and titration of sarbecovirus pseudoviruses

Pseudoviruses were produced by transfecting 293 T cells with spike protein expression plasmids via Lipofectamine 3000 (Invitrogen), followed by infection with the G*ΔG-VSV vector (Kerafast, Boston, MA, USA). Supernatants containing pseudoviruses were harvested at 24 and 48 h post-transfection, filtered through 0.45-μm membranes (Millipore), and stored at −80 °C. Pseudovirus titers were determined by infecting 293T-ACE2 cells with threefold serial dilutions of the pseudoviruses. The cells were incubated at 37 °C with 5% CO_2_ for 24 h. Bioluminescence signals were measured via the BriteLite Plus Reporter Gene Assay System (PerkinElmer, Waltham, MA, USA) in accordance with the manufacturer’s instructions.^37^

### In vitro pseudovirus neutralization assay

For pseudovirus neutralization assays, serum samples were initially diluted 1:30 and then subjected to threefold serial dilutions. Next, 50 μL of sarbecovirus pseudovirus (1.3 × 10^4^ 50% tissue culture infectious dose [TCID_50_]) was added to 96-well plates containing the diluted serum samples and incubated at 37 °C for 1 h. After incubation, 293T-ACE2 cells (3–5 × 10^4^ cells/well) were added, and the plates were incubated at 37 °C with 5% CO_2_ for an additional 24 h. Infectivity was assessed by measuring bioluminescence as described above. NT_50_ values were calculated via the Reed–Muench method.

### Phylogenetic tree construction

On the basis of the known ACE2-utilizing characteristics of sarbecoviruses described by Starr et al.^16^, we constructed a phylogenetic tree of 46 S protein amino acid sequences annotated with viral names, host species, and geographical origins (Supplementary Fig. 1). Among these, twenty-five sarbecoviruses from clades 1 and 3—representing lineages capable of ACE2 engagement—were further analyzed to construct phylogenetic trees on the basis of both full-length S protein and RBD amino acid sequences. Multiple sequence alignments for all the sequences were performed via MUSCLE software, and the alignment results were visualized via MEGA11 software. Maximum likelihood phylogenetic trees were constructed via RAxML software with 1,000 bootstrap iterations. These trees were edited and visualized via the online tool Evolview (https://www.evolgenius.info/evolview/#/treeview).

### Antigenic and phylogenetic distance analyses

To directly compare sequence-based phylogenetic relationships with empirically derived antigenic profiles, we constructed two complementary distance matrices. Antigenic distances were calculated from cross-neutralization NT50 values: for each serum–virus pair, the fold reduction in the NT50 relative to the homologous strain was log₂-transformed, and pairwise virus distances were symmetrized by averaging reciprocal measurements. Phylogenetic distances were derived from patristic branch lengths of a neighbor-joining tree reconstructed from full-length spike protein alignments via the BLOSUM62 substitution model.

The concordance between the antigenic and phylogenetic distances was assessed via Mantel tests (9999 permutations), which yielded correlation coefficients and significance values. To visualize clustering patterns, both distance matrices were subjected to principal coordinate analysis (PCoA). This approach applies double-centering to the squared distance matrix, followed by eigenvalue decomposition of the resulting matrix, yielding low-dimensional coordinates that best approximate the original distances. The first three principal coordinates were used to generate three-dimensional embeddings. To directly compare the antigenic and phylogenetic maps, Procrustes analysis was performed to align the two embeddings within a common coordinate system. The resulting Procrustes correlation coefficient (r) and disparity statistic quantified the degree of concordance and highlighted strains showing the greatest divergence between antigenic and evolutionary relationships.

### Statistical analysis

GraphPad Prism 8 (GraphPad, San Diego, CA, USA) was used for the statistical analyses. The data are presented as the means ± standard errors of the means (SEMs). Comparisons between two groups were performed via an unpaired two-tailed Student’s *t* test. For experiments involving more than two groups, one-way or two-way repeated-measures analysis of variance (ANOVA) followed by Dunnett’s within-subject multiple comparisons test was applied to account for interanimal variability. Significance thresholds were defined as follows: *P < 0.05, **P < 0.01, ***P < 0.005, ****P < 0.001.

## Supplementary information

Supplementary Materials

Supplementary Table

## Data Availability

All the data supporting the findings of this study are available within the article and Supplementary Materials. The original data for Figs. 1–4 have been deposited to Figshare: https://figshare.com/articles/figure/_i_Cross-neutralization_datasets_and_antigenic_maps_for_25_sarbecoviruses_i_/30608285?file=59530556.
