# Supplementary material for: Immunogenic relationship mapping supports a minimal-set trivalent vaccine strategy for broad sarbecovirus protection
Source: Signal Transduct Target Ther. 2026 Feb 9;11:48. doi: 10.1038/s41392-025-02565-5 (PMC12883627; doi:10.1038/s41392-025-02565-5)
Supplement: Supplementary file 1 — Supplementary Materials [file 41392_2025_2565_MOESM1_ESM.docx]

Supplementary Materials for

Immunogenic relationship mapping supports a minimal-set trivalent vaccine strategy for broad sarbecovirus protection

Yeqing Sun^1,2^, Ziqi Cheng^1,2^, Xi Wu^2^, Yunbo Bai^2^, Lina Zhao^1,2^, Hongyu Xiang^1^*, Weijin Huang^2^*, Jianhui Nie^2^*

Correspondence to: niejianhui@nifdc.org.cn

**This Word file includes:**

Supplementary Fig. 1 to 8


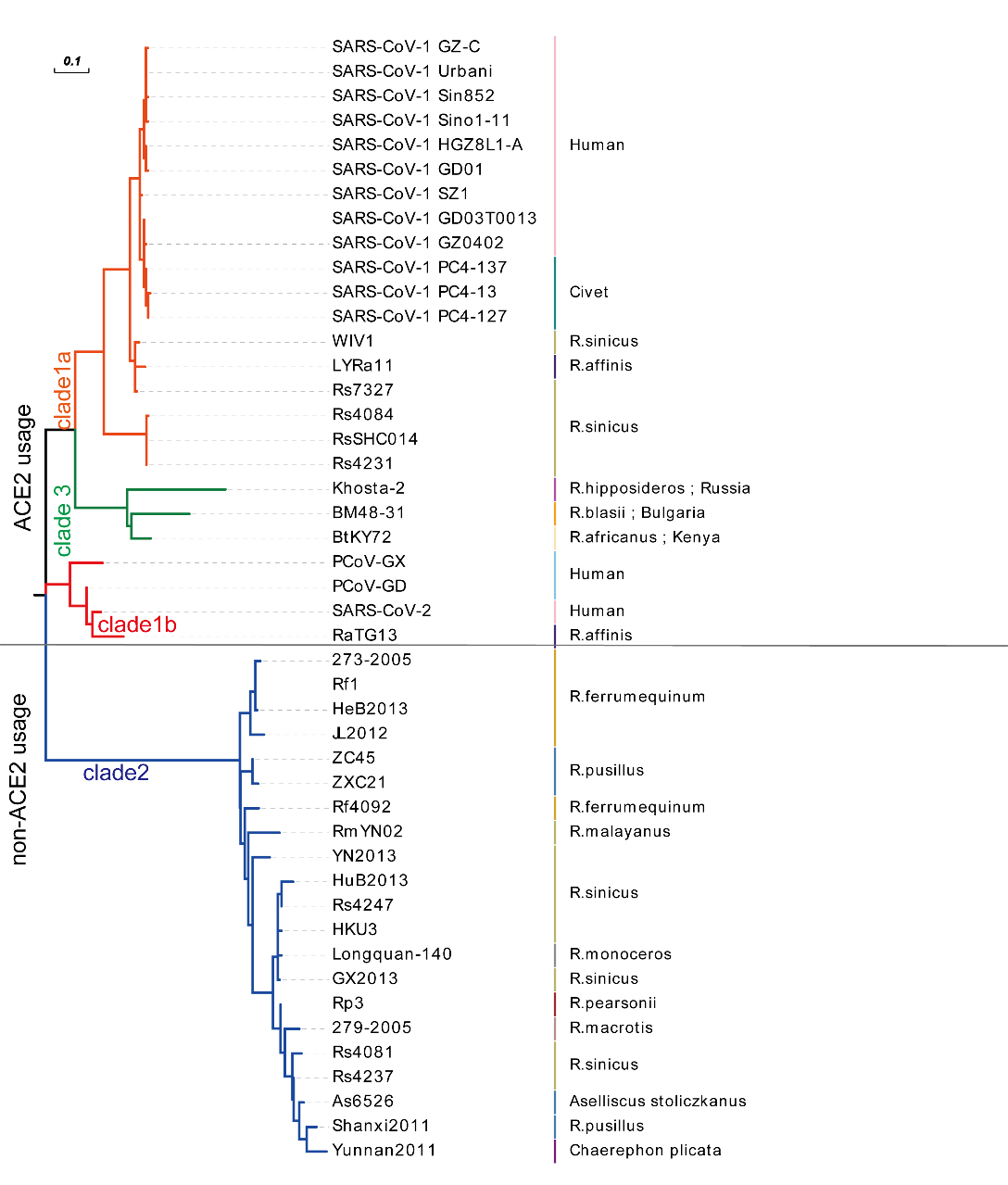


Supplementary Fig.1

**Phylogenetic relationships, host species, and geographic origins of representative sarbecoviruses.** Maximum-likelihood phylogenetic tree based on RBD protein sequences of representative sarbecoviruses, constructed using RAxML with 1,000 bootstrap replicates. The tree includes strains from clades 1a, 1b, 2, and 3, annotated with their host species and geographic origins (Asia unless otherwise indicated). This phylogenetic framework served as the basis for immunogen selection and for contextualizing antigenic and cross-neutralization analyses presented in the main text.


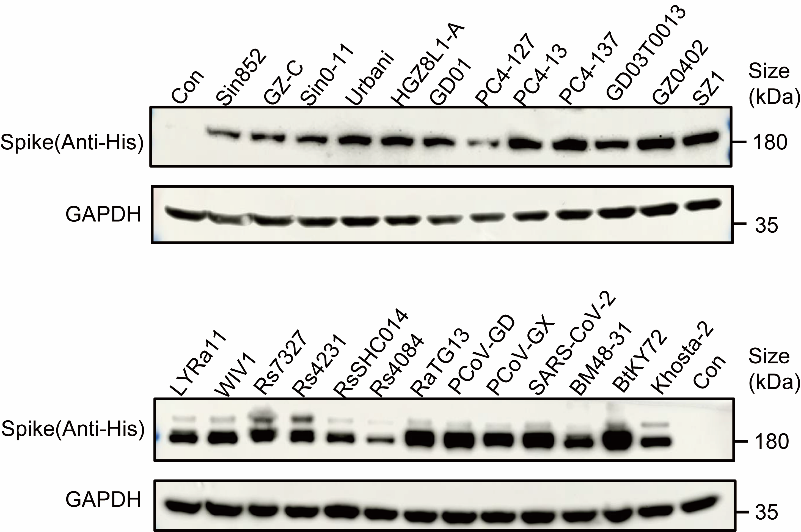


Supplementary Fig. 2

**Western blot analysis of sarbecovirus spike protein expression following DNA transfection.**
HEK293T cells were transiently transfected with plasmids encoding spike (S) proteins from 25 representative sarbecoviruses, each carrying a C-terminal His tag. Cells were harvested 48 hours post-transfection, lysed, and subjected to SDS-PAGE under reducing conditions. Spike expression levels were detected using a mouse anti-His monoclonal antibody (1:10000; AE003-ABclonal), while GAPDH was detected using a mouse anti-GAPDH antibody (1:30000; AC033-ABclonal) as an internal loading control


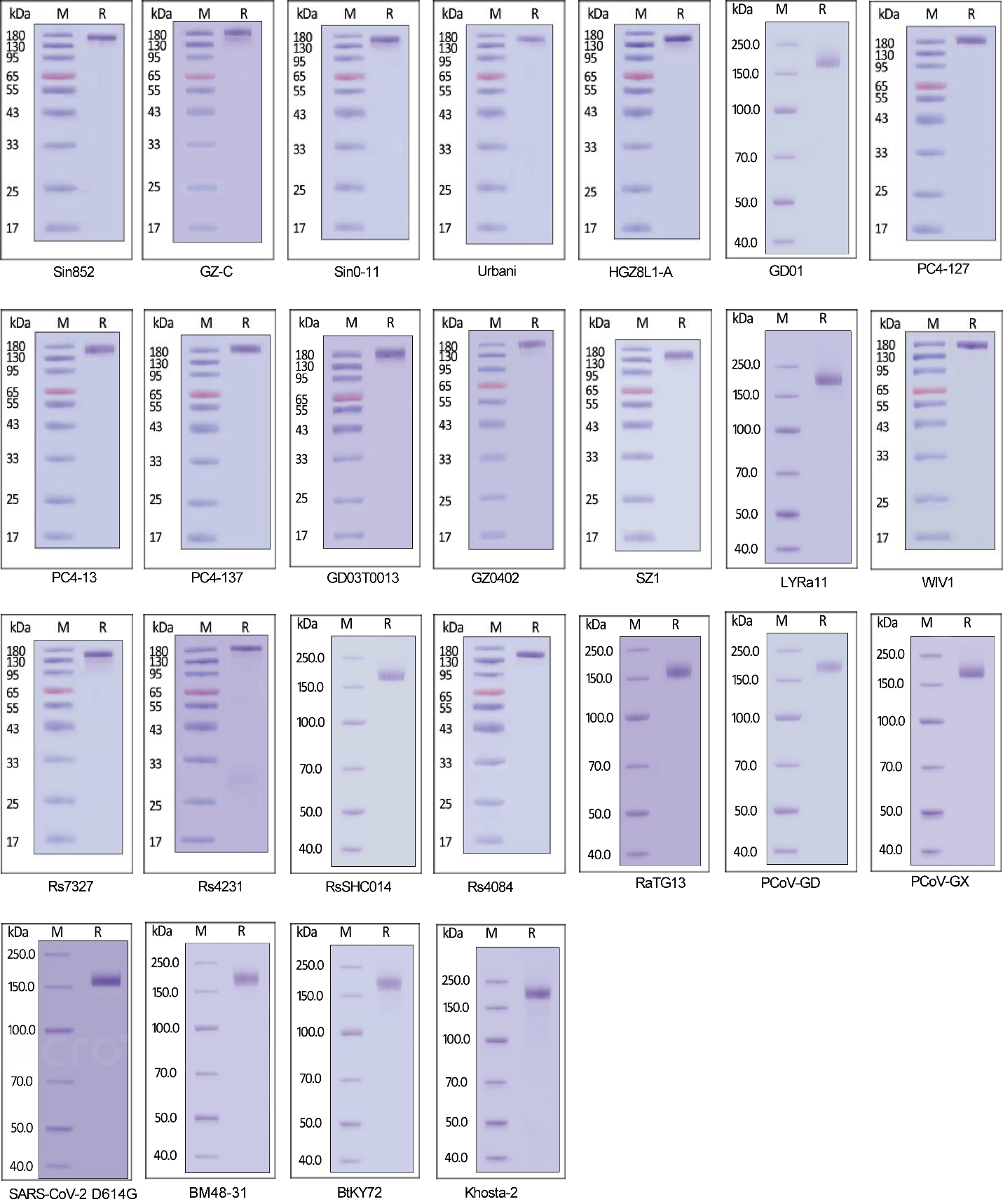


Supplementary Fig. 3

**Sarbecoviruses Spike Trimer, His Tag on SDS-PAGE under reducing (R) condition.** The gel was stained with Coomassie Blue. The purity of the protein is greater than 95%.


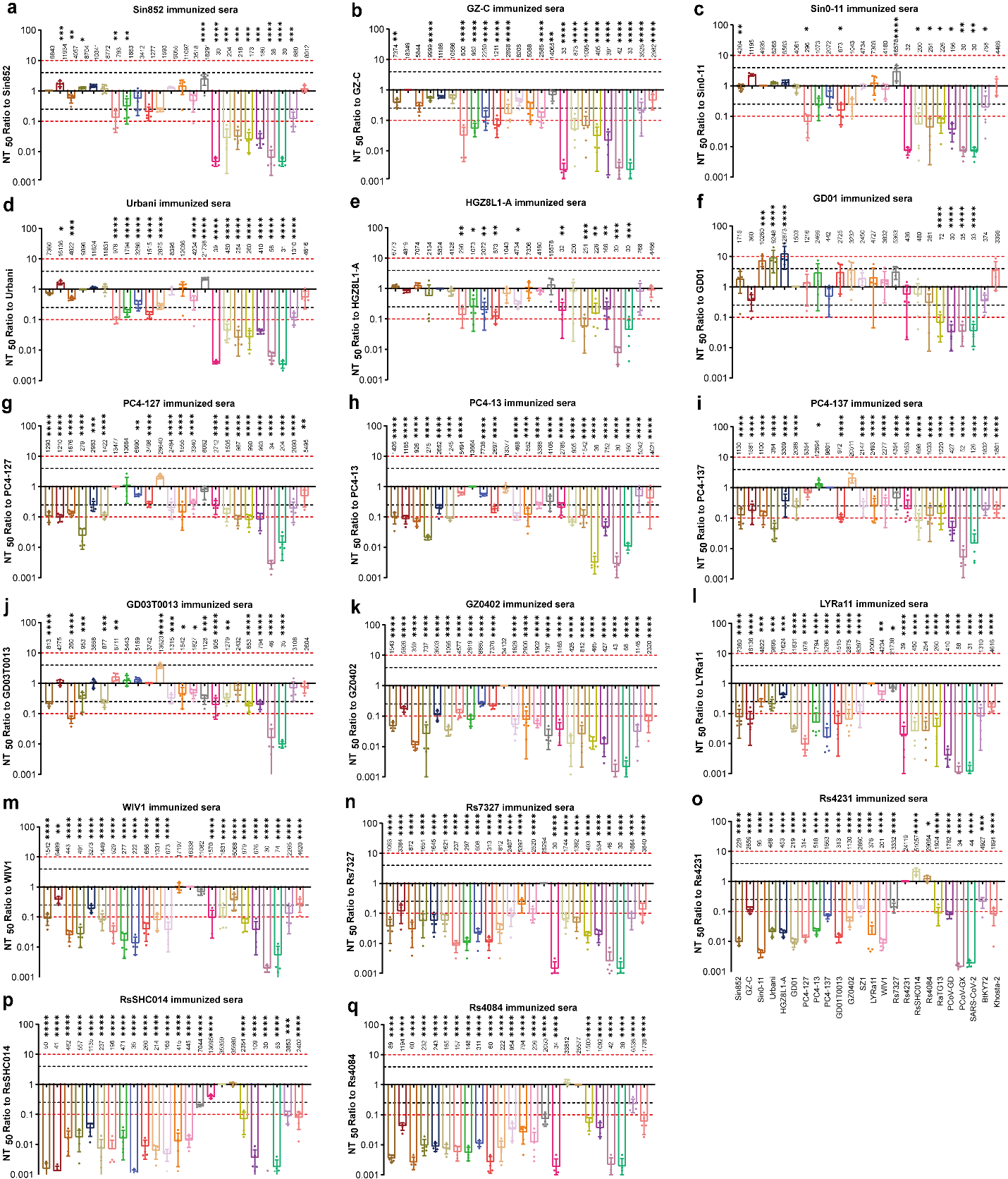


Supplementary Fig. 4

**Neutralization breadth against clade 1 sarbecoviruses**. **(a-q)** The x-axis shows the sarbecovirus pseudovirus strains, and the y-axis shows the NT50 values relative to those of the immunogen-matched strains. The black and red dashed lines denote 4-fold and 10-fold differences, respectively. Each dot represents one guinea pig (the mean of three technical replicates; n=5 per group). The results are presented as the means ± standard deviations (SDs). Two-way ANOVA and Dunnett’s multiple comparisons test were used for statistical analysis. Significant differences compared with the homologous immunization group are indicated by asterisks.


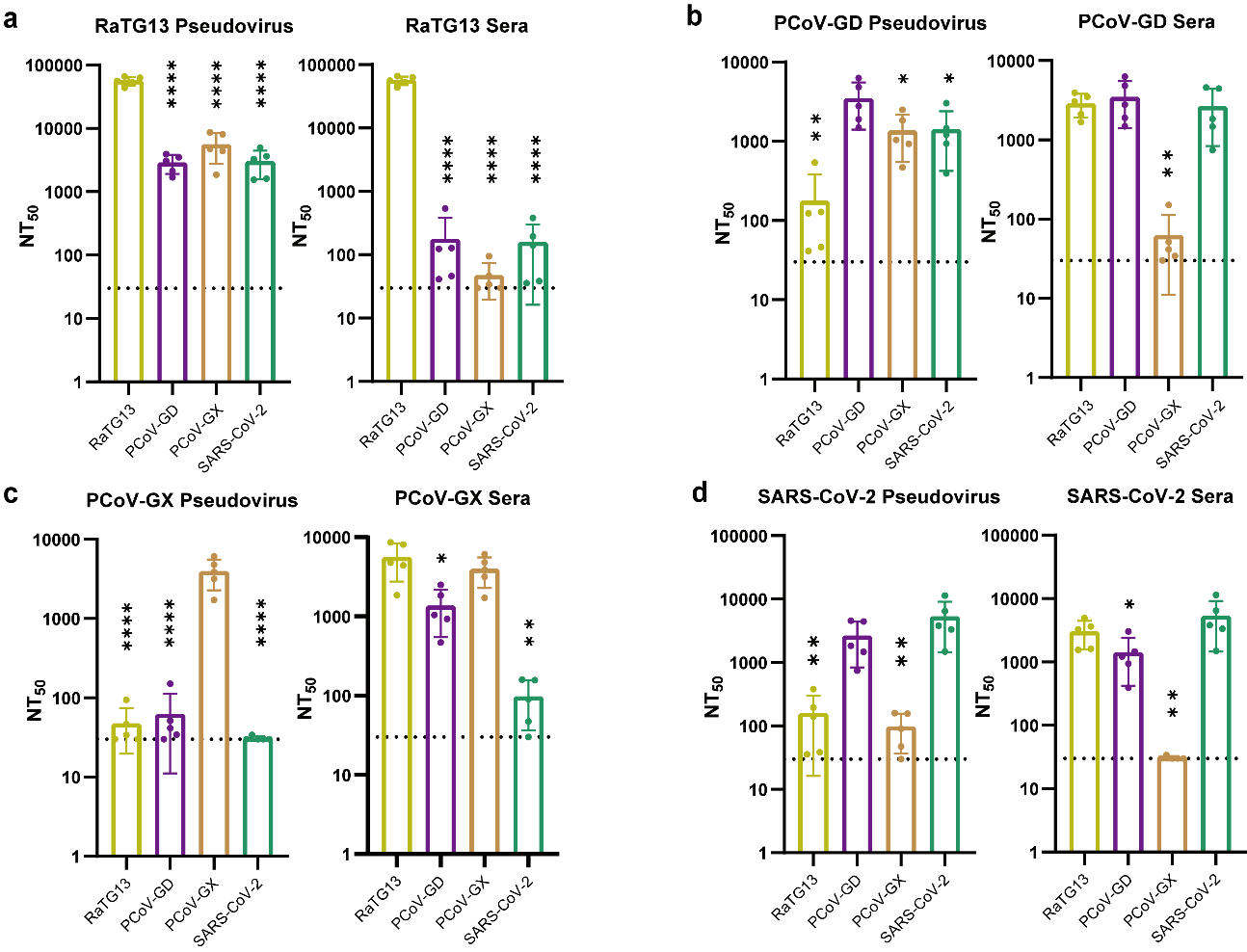


Supplementary Fig. 5

**Homologous and cross-neutralization profiles among clade 1b sarbecoviruses. (a-d).** Neutralization titers (NT₅₀) of reciprocal pseudovirus assays were measured between RaTG13, PCoV-GD, PCoV-GX, and SARS-CoV-2. Each panel shows the neutralization of a given pseudovirus (left) by heterologous sera and the reciprocal neutralization of sera from the same immunogen (right). Black dashed line indicates limit of detection. Each dot represents one guinea pig (the mean of three technical replicates; n=5 per group). Bars represent mean ± SEM of five animals. Two-way ANOVA and Dunnett’s multiple comparisons test were used for statistical analysis. Significant differences compared with the homologous immunization group are indicated by asterisks.


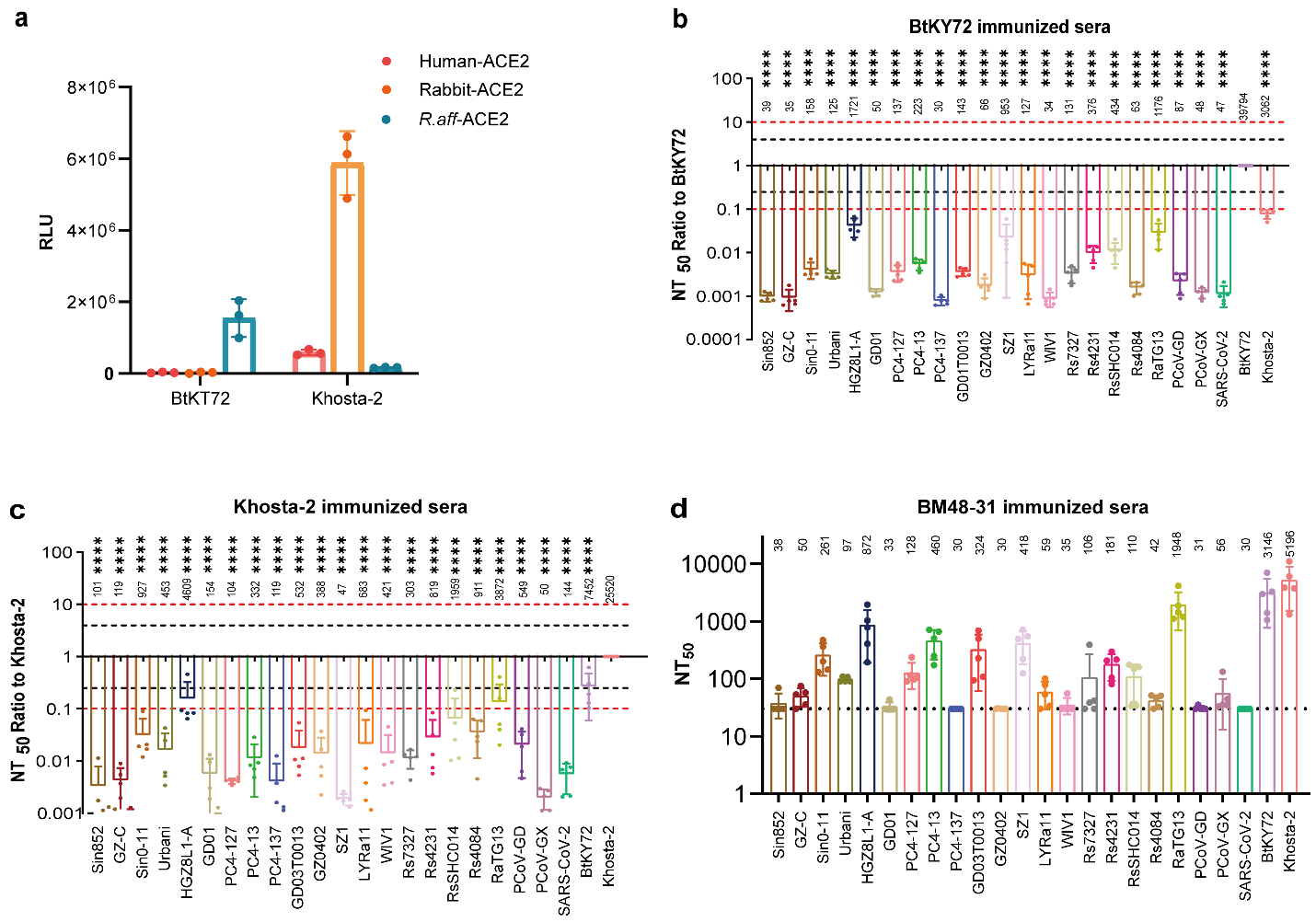


Supplementary Fig. 6

**Neutralization breadth against clade 3 sarbecoviruses**. **a.** For the pseudovirus neutralization assay of BtKY72 and Khosta-2, titration was performed using 293T cells overexpressing *R. affinis* ACE2 and 293T cells overexpressing rabbit ACE2, respectively. **(b-c).** The x-axis shows the sarbecovirus pseudovirus strains, and the y-axis shows the NT50 values relative to those of the immunogen-matched strains. The black and red dashed lines denote 4-fold and 10-fold differences, respectively. **d.** Neutralization sensitivity of BM48-31 immune serum against 24 sarbecovirus pseudoviruses other than itself. Black dashed line indicates limit of detection. Each dot represents one guinea pig (the mean of three technical replicates; n=5 per group). Bars represent mean ± SEM of five animals. Two-way ANOVA and Dunnett’s multiple comparisons test were used for statistical analysis. Significant differences compared with the homologous immunization group are indicated by asterisks.


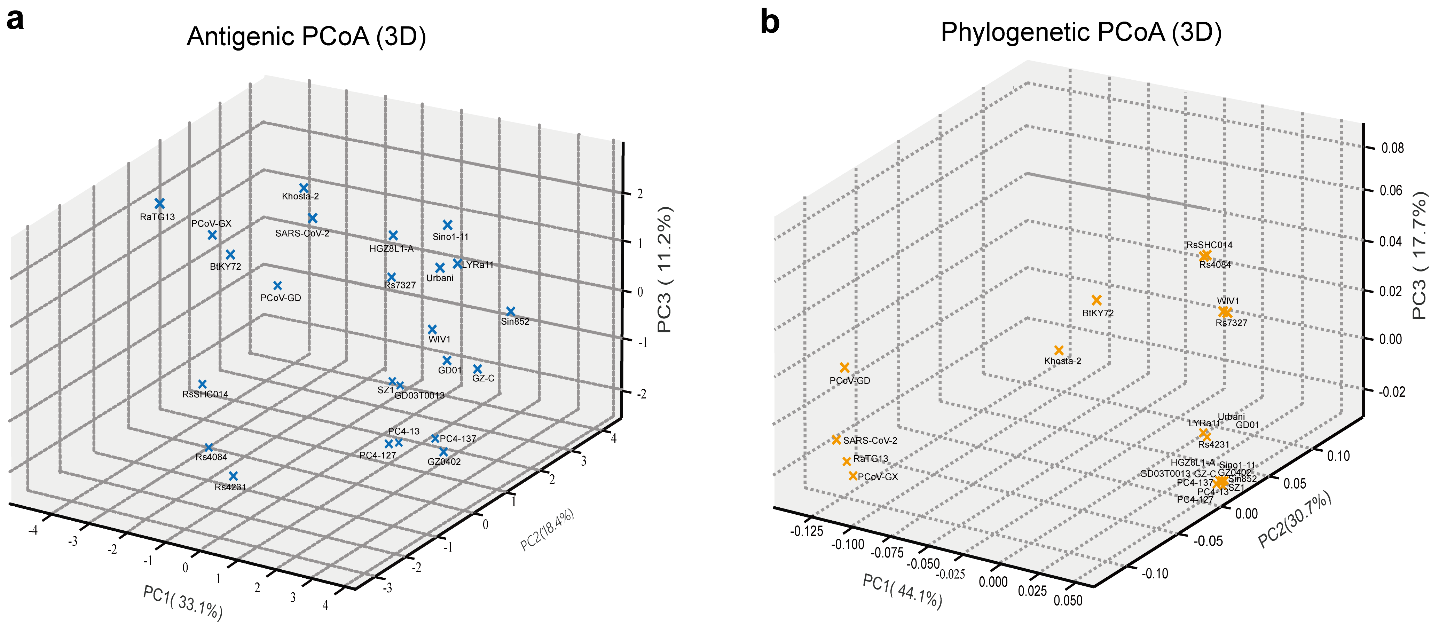


Supplementary Fig. 7

**a.** Three-dimensional principal coordinates analysis (PCoA) of antigenic and phylogenetic distances among 24 sarbecoviruses. Antigenic distances were calculated from cross-neutralization NT50 values by converting fold reductions relative to homologous titers into log₂ units and symmetrizing reciprocal measurements. **b.** Phylogenetic distances were obtained from patristic branch lengths of a neighbor-joining tree constructed from spike protein alignments using the BLOSUM62 substitution model. The first three principal coordinates are shown with variance explained indicated on the axes. Strain names are labeled for all data points.


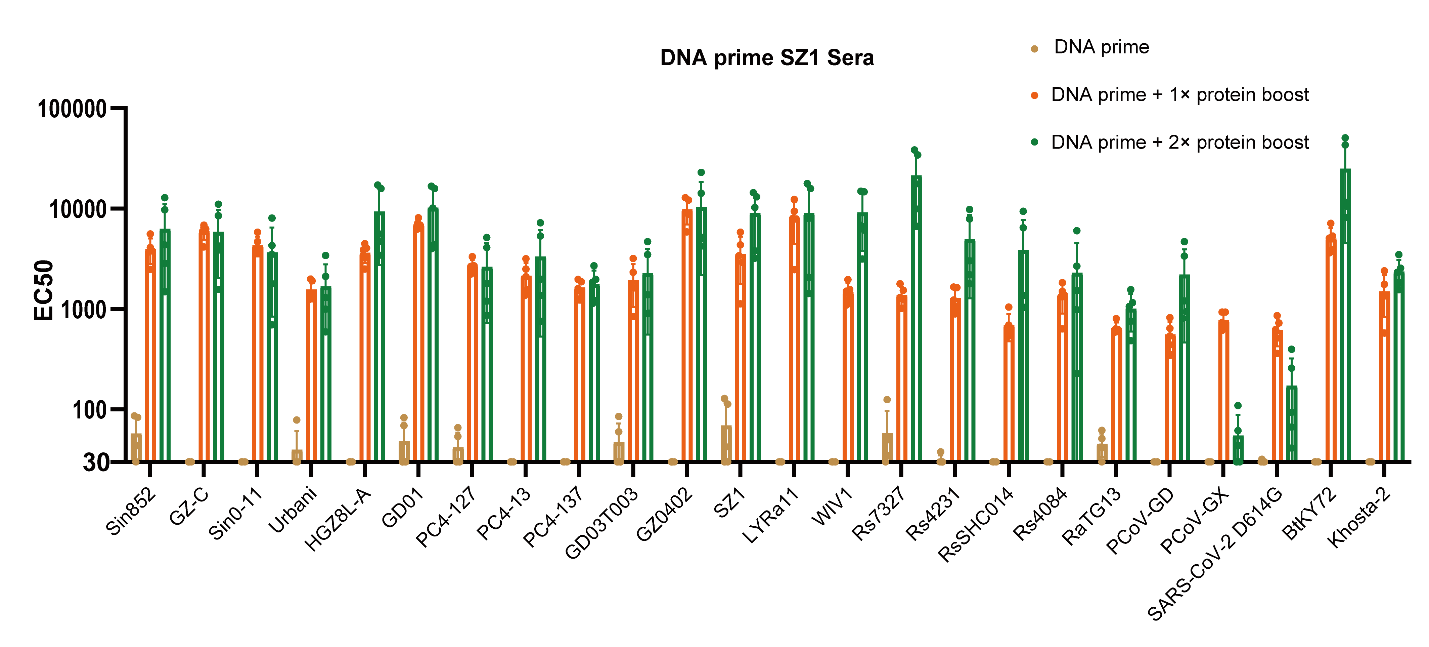


Supplementary Fig. 8

**Neutralization profiles across immunization timepoints.** Pseudovirus NT₅₀ values were measured in sera collected 14 days after the DNA prime and subsequent boosts. Each dot represents one guinea pig (the mean of three technical replicates; n=5 per group). The results are presented as the means ± standard deviations (SDs). Two-way ANOVA and Dunnett’s multiple comparisons test were used for statistical analysis.
